# P-1688. Prevalence of Mycoplasma hominis, Ureaplasma parvum, and Ureaplasma urealyticum along with antimicrobial susceptibility patterns of Mycoplasma hominis in vaginal samples from pregnant women in Japan

**DOI:** 10.1093/ofid/ofaf695.1862

**Published:** 2026-01-11

**Authors:** Hiroki Kitagawa, Kayoko Tadera, Mitsuyasu Ikeda, Norifumi Shigemoto, Hiroki Ohge

**Affiliations:** Hiroshima University Hospital, Hiroshima, Hiroshima, Japan; Hiroshima University Hospital, Hiroshima, Hiroshima, Japan; JA Hiroshima General Hospital, Hiroshima, Hiroshima, Japan; Hiroshima University Hospital, Hiroshima, Hiroshima, Japan; Hiroshima University Hospital, Hiroshima, Hiroshima, Japan

## Abstract

**Background:**

Information on *Mycoplasma hominis* resistance is currently limited in Japan. Therefore, this study investigated the antimicrobial susceptibility of *M. hominis* in clinical samples, along with the prevalence of *M. hominis* and *Ureaplasma* spp. in vaginal samples.

**Methods:**

Vaginal samples from pregnant Japanese women were analyzed for the presence of *M. hominis*, *U. parvum*, and *U. urealyticum* using conventional polymerase chain reaction. The susceptibility profile of 35 *M. hominis* strains stored at the Hiroshima University Hospital was evaluated using the MYCOFAST RevolutioN ATB+ kit, E-test, Eiken dry plates with urea-arginine LYO2 broth named the LYO2 broth method.

**Results:**

Out of 160 total vaginal samples, the prevalence of *M. hominis*, *U. parvum* and *U. urealyticum* was 4.4%, 28%, and 10%, respectively. Antimicrobial susceptibility testing using the MYCOFAST RevolutioN ATB+ kit revealed resistance rates of 17%, 11%, 0%, and 2.9% for levofloxacin, moxifloxacin, tetracycline, and clindamycin, respectively. Antimicrobial susceptibility testing results using the LYO2 broth method were consistent with the MYCOFAST RevolutioN ATB+ kit for levofloxacin, moxifloxacin, minocycline, and clindamycin. In addition, the minimum inhibitory concentrations (MIC) determined using E-test were similar to those of the MYCOFAST RevolutioN ATB+ kit.

**Conclusion:**

*M. hominis*, which is resistant to fluoroquinolones and clindamycin, was detected in clinical samples in Japan. In addition, we found that the LYO2 broth method was comparable to the MYCOFAST RevolutioN ATB+ kit in determining MICs. Therefore, the LYO2 broth method may be a reliable and convenient tool for determining the MICs of *M. hominis* in Japanese clinical settings.

**Disclosures:**

All Authors: No reported disclosures

